# Social isolation in the young and middle-aged patients with stroke: role of social support, family resilience and hope

**DOI:** 10.3389/fpsyt.2025.1499186

**Published:** 2025-02-12

**Authors:** Xiao Jia Wu, Ke Ke, Hui Liu, Shao Ping Zhan, Lei Wang, Juan Feng He

**Affiliations:** ^1^ Neurology Department, Renmin Hospital of Wuhan University, Wuhan, China; ^2^ General Department, Tong Ji Hospital, Tongji Medical College of Huazhong University of Science and Technology, Wuhan, China

**Keywords:** social isolation, stroke, the young and middle-aged patient, social support, family resilience, hope

## Abstract

**Background:**

Stroke is the leading cause of death and disability among adults in China. Social isolation in stroke survivors is a major public health concern across the globe. Social isolation is associated with social support, family resilience, and levels of personal hope, but how they interact to predict social isolation in the young and middle-aged stroke survivors remains unclear.

**Methods:**

Using cross-sectional design and convenience sampling method, a survey was conducted among 461 young and middle-aged stroke survivors. Perceived social support scale, General isolation Scale, Chinese version of Family Resilience Assessment Scale and Herth hope index were adopted to assess patients’ social, family and personal factors. SPSS 27.0 and AMOS 26.0 were used for descriptive analysis and structural equation modeling of the data.

**Results:**

The young and middle-aged stroke survivors had a high level of social isolation(49.57 ± 5.84). In the mediating effects model, social support could influence social isolation directly (95% CI -0.250, -0.061) or indirectly through family resilience (95% CI -0.136, -0.062) or patient hope level (95% CI -0.078, -0.017). In addition, Family resilience and hope had a significant chain mediating effect between social support and social isolation (95% CI -0.029, -0.006).

**Conclusions:**

Social support can have both direct and indirect effects on social isolation through the mediating factors of family resilience and hope. Clinicians and nurses can develop supportive interventions by taking integration of family and personal hope. On the one hand, resources can be directed to the individual patient, and on the other hand, the utilization of social support can be ensured by increasing family resilience and enhancing the coping capacity of family members and individuals.

## 1 Introduction

Social isolation refers to the active or passive isolation of individuals from the original community or social network relations, resulting in a decrease in the quantity and quality of interpersonal interactions and social activities (1, 2). In the past decades, more and more studies show people with high levels of social isolation present a higher prevalence of anxiety, depression, suicidality (3, 4) and cardiovascular disease (5), leading to delay recovery after disease (6). Social isolation is increasingly become a global public health problem. According to the domestic and international surveys, the prevalence of social isolation in the older adults population ranges from 5% to 43%, with a tendency to increase at a younger age (7, 8). Compared with the older adults, adolescents and young adults face changes in the quantity and quality of interpersonal interactions when they face major changes or transitions in their lives, such as sudden illnesses, economic decline, and leaving their parents (9, 10).

The World Health Organization (WHO) defines adults aged 18-60 as young and middle-aged people (11),which is the most productive years of the entire life and a period of rapid development of education, career and intimate relationships. However, globally, the incidence of stroke in young patients is on the rise, with high mortality and recurrence risk (12). In China, stroke is the leading cause of death and disability among adults, young and middle-aged stroke survivors (YMASS) accounts for about 50% of the total stroke population (13), and about 60% of stroke survivors have varying degrees of functional impairment (14), such as speech difficulties, dysphagia, limb dysfunction, sexual dysfunction, cognitive decline, resulting in those people being suddenly disengaged from normal life and work, and their interpersonal and social activities being negatively affected (15–17). Therefore, YMASS have to alter their family and social roles, and their economic burden and negative emotions have increased. Accordingly, some of these patients may actively distance themselves from previous relationships and reduce social activities, leading to social isolation (18–20). Previous studies have shown that social isolation not only reduces the adherence to therapeutic rehabilitation and increases the risk of functional impairment in post-stroke survivors (21), but is also a strong predictor of stroke recurrence or death (22). The existing literature mainly provides information on the status and influencing factors of social isolation in cancer survivors (23), maintenance hemodialysis patients (24), patients living with AlDS (25), patients with mental diseases (26) and patients with chronic diseases (27). Interventions improving social isolation are also focused on the older adults (28). The studies on social isolation in stroke survivors primarily focus on its impact on cardiovascular and cerebrovascular diseases as well as prognosis, with limited intervention research specifically targeting social isolation (21, 22). However, existing studies suggest that interventions such as rehabilitation, exercise training, cognitive rehabilitation, self-management programs, and vocational interventions can enhance social participation in stroke survivors, which, in turn, may help reduce social isolation (29–31). Therefore, there is a need to further explore the influencing mechanism of social isolation in YMASS, as well as to explore intervention directions to improve social isolation.

Social support refers to the perceived level of support from others or society, including tangible or intangible support such as information support, emotional support, financial support, and human resource support (32, 33). A study on social isolation of brain injury showed that social support was closely related to social relationships, and patients’ perceived/objective social support affects the quantity and quality of their social interactions (34, 35). When patients’ perceived social support fails to meet their expectations, feelings of isolation will arise (34). Good social support not only helps patients to establish a benign psychological state, but also helps them to choose healthy behaviors and positive coping styles, which is conducive to disease management (36, 37). In a survey on the level of social support for hospitalized patients, it was found that the score of social support for stroke survivors and the utilization of social support in China were in the middle to lower level (38–40). In China, family members are the primary caregivers for patients who need long-term care (41). Family resilience is defined as the ability of the family to cope with crises and stress, and to help family members recover, adapt and grow from difficulties (42). It has a positive impact on an individual’s physical and mental health, as well as on social adaptability (43, 44). As a protective factor, higher levels of social support are associated with better family resilience (45). Social support and family resilience are the driving force and source of hope for stroke survivors. Research indicates that strong social support can help patients maintain a positive outlook for the future. Empowerment interventions, positive psychology interventions and coping strategy interventions based on multidisciplinary teams can effectively enhance patients’ levels of hope by creating an environment that fosters hope (46, 47). Hope can help patients utilize social and family resources to positively influence themselves (48, 49). For these reasons, we propose that social support may have an indirect effect on social isolation via the mediating effects of family resilience and hope (Hypothesis 1).

On the one hand, family resilience can reflect the ability of family members to help patients overcome adversity and gain growth (42), and also reflect the degree of isolation of patients in the family environment (50). Nabors et al. believed that family resilience was related to anxiety, depression, and sense of worthlessness (51), and these negative emotions leading to a decline in patients’ willingness to social interaction are powerful predictors of social isolation (52). In China, family members are typically the primary caregivers for stroke survivors. Family support plays a crucial role in helping patients engage more effectively in rehabilitation, adopt positive coping strategies, and reduce negative emotions such as anxiety and depression (41). Hope is considered to be a strengthening factor (53). When patients believe family resilience is important and have better family resilience, they are more likely to use this resource to overcome current difficulties (54). Therefore, it is proposed that family resilience can predict social isolation directly and may have an indirect effect on social isolation via hope (Hypothesis 2).

International scholars have confirmed the same two elements of hope in different cultures: dynamic thinking (agency) and pathway thinking (pathways) (55). Snyder’s hope theory suggests that hope is the expectation of a positive future, which can improve the individual’s strong mental motivation (56). Individuals use hope as a positive coping strategy, rationalizing the use of their own resources, family resources, and social resources to cope with the financial burden, negative emotions and recovery from the disease (57, 58). A study on rehabilitation treatment of YMASS concluded that intervention based on the theory of hope could help patients improve their social interaction motivation and rehabilitation willingness, and promote healthy behavior (59). Guo et al. suggested that levels of hope can influence patients’ self-management behaviors and attitudes toward the future. Stroke survivors with lower levels of hope tend to have lower adherence to treatment and poorer social participation (46). Therefore, it is proposed that hope may have direct effect on social isolation (Hypothesis 3).

**Figure 1:**
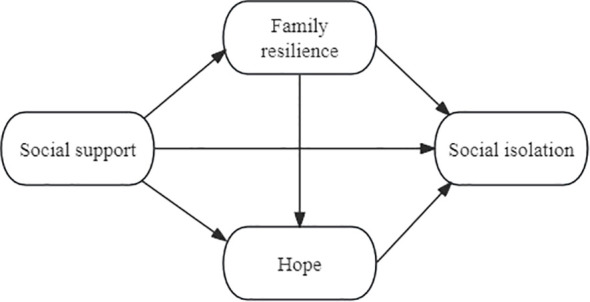
Proposed conceptual model of social isolation.

Based on the above Hypothesis 1, 2 and 3, as described above, this study intends to construct a Structural Equation Model (SEM) to examine the effects of social support, family resilience and hope on social isolation. The proposed conceptual model is shown in **Figure 1**. As far as the authors know, this is the first study to examine all these factors on social isolation.

## 2 Materials and methods

### 2.1 Setting and participants

This is a cross-sectional study to explore the mechanism of social isolation and intervention in YMASS. In this study, 506 patients hospitalized in Renmin Hospital of Wuhan University from March 2022 to February 2024 were selected as the subjects by convenience sampling method. Inclusion criteria: The patients should be 18-60 years old; The patients should be diagnosed with stroke by Magnetic Resonance Imaging (MRI) or Computed Tomography (CT) based on the Stroke Diagnostic Criteria (60, 61); The vital signs are stable and the course of disease is at least 3 months; There was no communication barrier; They all signed informed consent and volunteered to participate in this study. Exclusion criteria: Patients with previous psychiatric history; Patients were diagnosed with serious heart, kidney, liver diseases or malignant tumors; Patients who went through divorce, spouse loss or other major family accidents within six months.

A total of 461 YMASS completed the questionnaires. The self-report questionnaires used in this study were completed by participants on their cell phones through a questionnaire application. After each questionnaire was completed, the investigators reviewed the questionnaire immediately, and if there were any missing items or other problems, the questionnaire was verified and supplemented to the participants.

### 2.2 Measurements

Perceived Social Support Scale (PSSS) is used to measure the level of self-perceived social support. It is divided into three dimensions: family support, friend support, and other support, with four items each. The items are rated on a score of 1-7, with a total score of 12-84. The higher the score, the higher the level of social support. The scale has good reliability and validity in China (62).

The General isolation Scale (GAS), which was translated into Chinese by Wu Shuang and others, is used to measure the level of social isolation of patients. The scale includes 15 items in four dimensions: self-isolation (3 items), other-isolation (5 items), suspicion (4 items), and meaninglessness (3 items). The items are scored 1-4 points, with a total score of 15-60 points. The higher the score, the more serious the isolation. The scale has good reliability and validity in China (63).

Simplified Chinese version of the Family Resilience Assessment Scale (FRAS) was developed by Sixbey, translated and revised by Li Yuli, et al. (64). This scale is used to assess the level of family resilience in patients. There are 32 items in the scale, including three dimensions: family communication and problem solving, social resource utilization, and holding positive views. Each item uses a Likert 4 point scale. The total score ranges from 32-128 points. The higher the score, the higher the family resilience level. The scale has good reliability and validity in China (64).

The Herth hope index (HHl) was developed by Herth et al. (65) to assess the level of hope of patients. The scale has 12 items in total, which are divided into 3 dimensions: positive attitude (4 items: 1, 2, 6, 11), positive action (4 items: 4, 7, 10, 12), and intimate relationship (4 items: 3, 5, 8, 9). The scale is based on a Likert 4 point scale. Each item from “very opposed” to “very agreed” is scored 1-4 points in turn. Items 3 and 6 are reverse scoring items. The total score ranges from 12 to 48 points, with 12 to 23 points as low hope level, 24 to 35 points as medium hope level, and 36 to 48 points as high hope level. The scale has good reliability and validity in China (66).

### 2.3 Ethical considerations

Based on the principle of voluntary participation and withdrawal at any time, participants signed informed consent before the survey. Questionnaires were recorded anonymously and stored separately to protect the privacy of participants. The study protocol (No: WDRY2021K155) was approved by the Ethics Committee of Renmin Hospital of Wuhan University. The investigation was carried out in accordance with the Declaration of Helsinki.

### 2.4 Data analysis

Epidata 3.1 software was used for data entry. IBM SPSS 27.0 and IBM SPSS AMOS 26.0 software were used for data analysis. Measurement data were described as mean ± standard deviation and count data were described as frequency and percentage. The relationship between social support, family resilience, hope and social isolation was analyzed using Pearson correlation analysis. Based on Hypotheses 1, 2 and 3, the structural equation modeling was constructed using AMOS 26.0 software. The maximum likelihood method was applied to estimate the parameters of the structural equation model. The model was modified according to the correction index. According to conventional criteria, χ2/df < 3, RMSEA < 0.08, CFI and TLI > 0.90 indicate acceptable criteria (67). Bias-corrected percentile Bootstrap method (5000 replicated samples) was used for mediation effect tests. All tests were two-tailed and differences were considered statistically significant at P < 0.05.

## 3 Results

### 3.1 Sociodemographic data of the sample

A total of 506 questionnaires were distributed and 461 valid questionnaires were recovered, with a valid recovery rate of 91.1%. A total of 461 YMASS were included, with a mean age of 49.69 ± 7.74 years, including 301 males (65.4%) and 160 females (34.6%). Specific results are shown in **Table 1**.

**Table 1:** Sociodemographic data of young and middle-aged stroke survivors (N=461).

Variables	Number of cases [n (%)]	Variables	Number of cases [n (%)]
Gender		History of stroke	
Male	301 (65.4)	No	212 (46.0)
Female	160 (34.6)	Yes	249 (54.0)
Residence		History of chronic disease	
Towns	365 (79.1)	No	169 (36.7)
Rural	96 (20.9)	Yes	292 (63.3)
Educational level		Occupational status	
Primary and below	55 (11.9)	On job	274 (59.5)
High l/Secondary school	136 (29.6)	Unemployment	27 (5.8)
College and above	270 (58.5)	Others	160 (34.7)
Marital status		Type of health insurance	
Unmarried	60 (13.1)	Basic health insurance	159 (34.6)
Married	364 (78.9)	Medical insurance for urban employees	211 (45.7)
Divorced	22 (4.7)	Commercial insurance	14 (3.1)
Widowed	15 (3.3)	Combined insurance	77 (16.6)
Primary caregivers		Average family income (Yuan)	
Spouse	221 (48.0)	<3000	104 (22.6)
Children	117 (25.3)	3000~5000	200 (43.5)
Parents	75 (16.2)	5000~8000	135 (29.2)
Others	48 (10.5)	>10000	22 (4.7)

### 3.2 Correlation of study variables

Correlations between the variables of social support, family resilience, hope, and social isolation are shown in **Table 2**. A negative correlation was found between social isolation and social support (r=-0.360), family resilience (r=-0.410) and hope (r=-0.349), both at the 1% significance level. Social support was positively correlated with family resilience (r = 0.354) and hope (r = 0.377), both showing 1% significance level. Family resilience was positively correlated with hope (r = 0.396) with a significance level of 1%.

**Table 2:** Correlation analysis of social support, family resilience, hope and social isolation in the young and middle-aged stroke patients (N=461).

Variables	± SD	SS	FR	H	SI
SS	38.16 ± 6.42	1.000			
FR	94.48 ± 8.15	0.354**	1.000		
H	32.64 ± 6.31	0.377**	0.396**	1.000	
SI	49.57 ± 5.84	-0.360**	-0.410**	-0.349**	1.000

SS, Social support; FR, Family resilience; H, Hope; SI, Social isolation.

**P<0.01; *P<0.05.

### 3.3 Regression coefficients and mediating effects of all paths in the model

**Figure 2:**
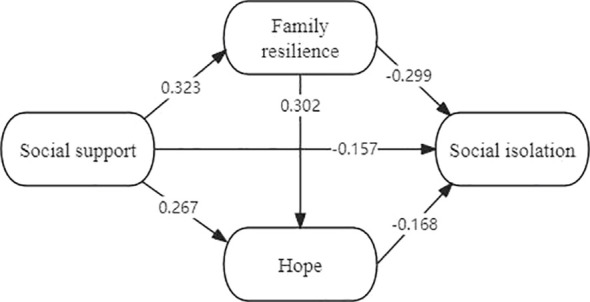
Structural equation model of social isolation.

The initial structural equation model (SEM) obtained according to the assumptions is shown in **Figure 2**. The fitting result of the model were as follows: χ2/df=3114.892/2408 = 1.294, P<0.01, RMSEA=0.025, CFI=0.972, TLI=0.971, indicating an acceptable goodness of fit.

As the results indicated, social support had a direct effect on social isolation and an indirect effect on social isolation via family resilience and hope, which supported Hypothesis 1. Family resilience had a direct effect on social isolation and an indirect effect on social isolation via hope, which supported Hypothesis 2. Hope had a direct effect on social isolation, which supported Hypothesis 3. The standardized parameters of each path are shown in **Table 3**, and the final SEM is shown in **Figure 2**.

**Table 3:** Regression coefficients and mediating effects of all paths in the final model.

Relationship	Estimate	S.E.	P-Value
SS→FR	0.323	0.022	<.0001
SS→H	0.267	0.016	<.0001
SS→SI	-0.157	0.033	<.0001
FR→SI	-0.299	0.069	<.0001
FR→H	0.303	0.033	<.0001
H→SI	-0.168	0.100	<.0001

SS, Social support; FR, Family resilience; H, Hope; SI, Social isolation.

### 3.4 The influence of variables in the model on social isolation

The effect of the influencing factors on social isolation is shown in **Table 4**, which include the total, direct and indirect effects.

**Table 4:** Influence of variables on SI in the final model.

Variables		SS	FR	H
SI	Direct effect	-0.157	-0.157	-0.168
	Indirect effect	-0.097	-0.045	/
	Total effect	-0.253	-0.201	-0.168

SS, Social support; FR, Family resilience; H, Hope; SI, Social isolation.

## 4 Discussion

This study explored the effect of social isolation on young and middle-aged stroke survivors from the social support, family resilience and individual hope levels. We tested the effects of family resilience and individual hope level as chain-mediated variables on social support and social isolation. The results of the mediation effects analysis indicated that social support can independently predict social isolation and indirectly predict social isolation through family resilience and hope. On one hand, we analyze whether creating a supportive family and social environment can protect stroke survivors from the negative effects of social isolation. On the other hand, we explore whether the combined influence of family resilience, hope, and social support can activate the internal resources of stroke survivors, encouraging active participation and reducing the motivation for self-imposed social withdrawal. This provides a basis for a better understanding of the social, family, and individual factors associated with social isolation in YMASS, thus may offering more targeted clues for interventions.

### 4.1 Effect of hope on social isolation

Stroke places a large financial and psychological burden on patients, and how patients cope with post-stroke outcomes is related to disease recovery and quality of life (20). Hope exists at every stage of life and is an individual’s expectation and motivation to achieve a stakeholder goal (68). After stroke, YMASS may experience difficulties such as physical dysfunction, interruption of schooling and career, changes in intimate relationships, and increased family burdens (15, 16). Hope provides patients with a sense of optimism about the future, so that when they face difficult situations, they are inclined to choose a positive way of coping, mobilize available resources to change the status or even take the initiative to seek help, instead of being trapped in negative emotions and alienating themselves from their friends and society (69, 70). Rehabilitation of stroke patients requires a long period of time and adequate resources, so it is especially important to assess and take measures to maintain and improve the patient’s level of hope during the long recovery process.

### 4.2 Effect of family resilience on social isolation

This study holds that family resilience had a direct impact on social isolation. In China, the primary caregivers of stroke patients come from their families (41). Family dysfunctioning and caregivers’ overburden may negatively affect patients’ psychological status and self-management ability, which in turn reduces patients’ subjective experience of the quality of family relationships and interpersonal relationships (71). In addition, family resilience can indirectly affect social isolation through hope. Positive psychology believes that family resilience is a kind of force that helps families to realize healthy adjustment and recovery from major stressors (72). Good family resilience can enhance patients’ hope and psychological resilience (49), and improve patients’ motivation and ability to solve problem (73). In the process of problem solving, patients will take the initiative to increase contact and communication with people, society, and the environment, and can detach from social isolation. A follow-up study based on the dichotomous perspective of stroke patients and their caregivers (74)found that although family resilience of patients and caregivers tended to increase over time during a 6-month follow-up period, the overall level was low, and the level of patient-perceived family resilience was consistently lower than that of caregivers in the same group of families. Therefore, while exploring how to improve family resilience in stroke patients, it is also necessary to focus on the mechanisms by which family resilience acts on patients, to reduce patient-caregiver variability in perceptions of family resilience from patients’ intrinsic traits, and to improve patients’ recognition and utilization of family strengths. To the best of our knowledge, this study is the first to explore the role of personal hope in family resilience and social isolation, and provides a new direction for clinical development of interventions that use family strengths as a starting point for improving patient outcomes.

### 4.3 Effect of social support on social isolation

Social support has a direct impact on social isolation, which is consistent with previous research (34). Mahon et al. (75) believe that social support can influence patients’ health behavior by providing information and guidance in social relations. Different from other populations, stroke patients with dysfunction have limited ability to establish or maintain social relations (76, 77), for example, patients with aphasia after stroke have limited ability to communicate with others, and patients with hemiplegia after stroke cannot leave home to participate in social activities, which may result in decreased resources for social support and an increased risk of social isolation (78). Compared with friends and social relationships, family relationships of stroke patients are relatively stable (79). However, due to the longer course of disease and greater dependence on the family, there is a gradual increase in stress within the patient’s family, the sustainability of family resources is facing greater challenges (80).

Social support can be provided in many forms, usually divided into emotional support, instrumental support, information support, financial support and evaluation support (81). Stroke survivors’ needs for social support are highly variable at different stages of the disease, and there is no evidence that comprehensive interventions can meet the needs of all patients (82). Padberg et al. found that in Germany, stroke inpatients pay more attention to home-based topics after discharge, while home-based patients pay more attention to mutual aid groups, secondary prevention and outpatient treatment (82). A survey on the needs analysis of stroke patients in China showed that patients in the acute phase had greater needs for disease progress and supportive emotions, while patients in the stable phase showed concerns and worries about rehabilitation and exercise programs, cost reimbursement, and long-term caregivers, and patients in the discharge preparation period expressed the need for health guidance, secondary prevention, home follow-up, and help from rehabilitation institutions. Home rehabilitation patients paid more attention to disease knowledge, self-care ability, rehabilitation plan, follow-up assessment, returning to work, acupuncture and supportive feelings (83, 84). Based on the above research, we need to integrate all resources (including human resources, such as medical and rehabilitation personnel, counterpart community chronic disease management departments, social workers, patients’ family members; economic support, such as medical insurance policies, medical assistance funds; material resources, such as home modifications, means of transport, etc.) to develop systematic intervention strategies, and provide intervention according to the needs of patients at different stages or the needs of the same patient at different stages.

Social support can indirectly affect social isolation through family resilience and hope. Multidimensional social support can improve family’s coping ability, promote effective communication between family members and increase family resilience (50). Based on past experiences, perceptions, and current disease state, patients hold a certain level of hope about the current problems they face (85), and when they perceive adequate social support and good family resilience, they will increase their confidence in overcoming difficulties and enhance their positive mindset about their future life (69). Additionally, when patients approach problems in a positive way, positive interaction with health care professionals, family, friends, and the community can be established to improve the number and quality of social contact and interpersonal interactions (86). However, Palmer et al. (87) found that when the majority of family resources and social support were focused on the patient, the burden of caregivers was not alleviated, but family resilience was reduced, which was not conducive to the effective use of social support. Therefore, we need to consider both the needs of young and middle-aged stroke patients and caregivers when providing supportive interventions to ensure that family resources and social support make the most of the impact.

Given the prolonged course of recovery for stroke survivors, the levels of family resilience, hope, and social support tend to fluctuate over time. As such, interventions for these patients should not be limited to a single time point, but rather span multiple phases of recovery. Based on the findings, we recommend a long-term follow-up approach that includes continuous, dynamic assessments of patients’ individual levels of hope, family resilience, and social support. This would allow healthcare providers to tailor interventions based on the evolving needs of stroke survivors, adjusting resource allocation accordingly. Furthermore, as hope and family resilience play a mediating role in the relationship between social support and social isolation, we suggest that intervention strategies should target both the individual patient and their family. By addressing both psychosocial factors simultaneously, such interventions could more effectively mitigate social isolation and enhance overall recovery.

## 5 Limitations

This study has several limitations. First, data were collected using a convenience sampling method from a single hospital in Wuhan, Hubei Province, China, which may limit the generalizability of the findings to other regions or settings. Second, the self-reported nature of the data collection procedure may introduce bias, as participants’ responses could be influenced by subjective perceptions or recall inaccuracies. Third, while sociodemographic variables such as education level and marital status are known to correlate with factors like social support and social isolation, these variables were excluded from the current analysis to avoid multicollinearity and maintain model stability. Sensitivity analyses in future research could further explore their potential impact and validate alternative models. Finally, residual impairments may affect the degree of social isolation and the dynamic interactions among hope, family resilience, and social support. Although the inclusion criteria accounted for functional impairments of stoke survivors, detailed assessments were not conducted due to logistical constraints. Incorporating comprehensive evaluations of impairment levels in future studies could provide deeper insights into their role in social isolation and related psychosocial factors.

## 6 Conclusion

In this study, YMASS had a high level of social isolation (49.57 ± 5.84). Social isolation is a strong predictor of recurrence, treatment, and rehabilitation outcomes in cerebrovascular disease (22), especially in YMASS, who are more sensitive to social relationships and role changes (18–20). Therefore, it is essential to emphasize the early assessment of social isolation in young and middle-aged stroke survivors and to give targeted interventions. This study found that social support can directly affect social isolation, and can also indirectly affect social isolation through family resilience or patients’ hope level. In addition, family resilience and hope play a significant chain mediating role between social support and social isolation. These findings may provide new perspectives for the development of social isolation interventions, namely that social support can integrate family resilience and individual hope levels. On the one hand, resources are directly used for the individual patient, and on the other hand, the utilization of social support can be ensured by increasing family resilience and enhancing the coping capacity of family members and individuals. In order to provide more effective interventions for patients, the mechanism of the role of protective factors under the caregiver-patient dichotomy perspective needs to be further explored.

## Data Availability

The raw data supporting the conclusions of this article will be made available by the authors, without undue reservation.
